# Commentary: Rapid clinical identification of vulnerable older adults in acute and emergency care: narrative review of short geriatric screening tools

**DOI:** 10.3389/fmed.2026.1969593

**Published:** 2026-10-29

**Authors:** Pedro K. Curiati, Thiago J. Avelino-Silva, Márlon J. R. Aliberti

**Affiliations:** 1 Geriatric Emergency Department Research Group (ProAGE), Hospital Sírio-Libanês, São Paulo, Brazil; 2 Geriatric Center for Advanced Medicine, Hospital Sírio-Libanês, São Paulo, Brazil; 3 Laboratorio de Investigacao Medica em Envelhecimento (LIM-66), Servico de Geriatria, Hospital das Clinicas HCFMUSP, Faculdade de Medicina, Universidade de Sao Paulo, São Paulo, Brazil; 4 Division of Geriatrics, University of California, San Francisco, CA, United States

**Keywords:** acute care, emergency medicine, geriatric assessment, hospital medicine, prognosis

## Introduction

We read with great interest the recent narrative review by Cešljarac et al. (1), which provides a timely and pragmatic overview of brief geriatric screening tools for emergency departments and acute care settings. We agree with the authors’ proposed tiered, two-step approach, in which rapid screening identifies vulnerable older adults who may benefit from subsequent multidomain assessment and targeted interventions. However, as Cešljarac et al. state in their methodology, ‘clinical relevance and clarity were prioritized over exhaustive coverage’. While we agree with this pragmatic approach, prioritizing brevity may inadvertently lead to the omission of composite tools. We respectfully suggest that including the PRO-AGE score, developed in Brazil in 2020 (2), is highly clinically relevant and warrants consideration within their proposed framework.

The PRO-AGE score is a brief multidomain screening instrument designed for rapid risk stratification of older adults in emergency and acute care settings. It integrates key geriatric domains—Physical impairment, Recent hospitalization, Older age, Acute mental alteration, Getting thinner, and Exhaustion—into a single bedside assessment (2). The score ranges from 0 to 8, combines brief patient- or proxy-reported information with routinely available clinical data, and can be calculated in typically less than 2 min. As such, it aligns with the strict time constraints and rapid clinical workflows described by Cešljarac et al. (1). Beyond its brevity, capturing multidomain constructs during this rapid assessment may enrich the initial screening step without replacing the need for a subsequent comprehensive evaluation.

The evidence supporting PRO-AGE has evolved from its original derivation to multicenter validation across emergency care settings, providing consistent evidence of its predictive performance (2–4). Across these investigations, PRO-AGE consistently demonstrated good discrimination for clinically meaningful outcomes, including hospital admission, prolonged length of stay, short-term mortality, and post-discharge functional decline. Importantly, head-to-head comparisons demonstrated incremental prognostic value over established screening instruments such as ISAR and FRAIL (2–4), and incorporating PRO-AGE into illness-acuity measures (e.g., NEWS) further improved mortality risk stratification among hospitalized older adults with COVID-19 (4). Collectively, these studies encompassed over 7,800 older adults. While this constitutes a robust evidence base, the current validation remains geographically concentrated primarily in Brazil, alongside a smaller multicenter cohort in the United States (Table 1). To further establish its broader international applicability beyond the Americas, additional external validation studies are currently underway in the European Union and the United Kingdom.

**Table 1:** Summary of PRO-AGE validation studies.

Study	Setting & population	*N*	Predicted outcomes	Key performance metrics
Curiati et al. (2) *Ann Emerg Med*	Geriatric ED (Brazil). Patients ≥ 70 years.	5,025	Hospital admission; Prolonged length of stay (>7 days); In-hospital death.	AUC 0.74 (admission); AUC 0.79 (prolonged stay); AUC 0.89 (death). Outperformed ISAR and FRAIL (NRI 16%–46%).
Aliberti et al. (4) *Age & Ageing*	Tertiary University Hospital, COVID-19 wards (Brazil). Patients ≥ 50 years.	1,428	30-day and 60-day mortality.	AUC 0.70 (60-day mortality). Outperformed ISAR (AUC 0.62). Improved prediction when added to illness acuity (NEWS).
Cohen et al. (3) *JAGS*	Multicenter Federated Study (US and Brazil). ED admissions ≥65 years.	1,390	90-day mortality; 90-day functional decline (ADL/IADL).	Upper PRO-AGE group strongly predicted 90-day death (US HR = 11.76; BR HR = 12.29) and new ADL disability (US HR = 2.08; BR HR = 1.70).

ADL, activities of daily living; AUC, area under the receiver operating characteristic curve; BR, Brazil; COVID-19, coronavirus disease 2019; ED, emergency department; FRAIL, fatigue, resistance, ambulation, illnesses, and loss of weight; HR, hazard ratio; IADL, instrumental activities of daily living; ISAR, Identification of Seniors at Risk; NEWS, National Early Warning Score; NRI, net reclassification improvement; PRO-AGE, physical impairment, recent hospitalization, older age, acute mental alteration, getting thinner, and exhaustion; US, United States.

## Discussion

Cešljarac et al. appropriately conclude that no single screening instrument simultaneously achieves high sensitivity and specificity, and that the effectiveness of geriatric screening depends on its integration into routine clinical workflows linked to actionable interventions (1).

Rather than blurring the traditional distinction between rapid screening and multidomain evaluation, PRO-AGE may enhance the first step of this framework. It does not replace the need for a comprehensive assessment; instead, by capturing specific multidomain deficits (e.g., flagging mobility or cognitive issues) during the rapid initial screen, it may allow clinicians to intelligently target and tailor the second step of the evaluation.

Within the proposed framework, PRO-AGE may serve as an initial risk-stratification tool. A high score can prompt focused assessment of cognition, mobility, nutrition, medication use, and patient priorities, followed by interventions matched to the deficits identified. As emergency departments increasingly adopt age-friendly models of care, there is growing demand for screening tools that combine efficiency with clinically meaningful multidomain information. Within this evolving landscape, PRO-AGE represents an evidence-based option supported by derivation, external validation, and international multicenter validation studies (2–4). We believe that its inclusion alongside established instruments would provide readers with a more comprehensive overview of contemporary strategies for rapid geriatric risk stratification in acute care.

In agreement with Cešljarac et al. (1), we acknowledge that no screening tool is without limitations. The PRO-AGE score relies on patient or proxy recall for items such as unintentional weight loss and acute functional decline, which may be difficult to ascertain in overcrowded emergency departments if a reliable informant is absent. Furthermore, prioritizing high sensitivity for vulnerability inevitably results in moderate specificity, meaning PRO-AGE is best utilized precisely as Cešljarac et al. recommend: as an initial trigger rather than a definitive diagnostic tool.
